# Supplementary material for: Dietary DHA Enhanced the Textural Firmness of Common Carp (Cyprinus carpio L.) Fed Plant-Derived Diets through Restraining FoxO1 Pathways
Source: Foods. 2022 Nov 11;11(22):3600. doi: 10.3390/foods11223600 (PMC9689754; doi:10.3390/foods11223600)
Supplement: Supplementary file 1 [file foods-11-03600-s001.zip › foods-1972570-supplementary.pdf]

Table S1 The primer sequences used for qRT-PCR.

Transcript	Primers	Sequence 5'-3'
**Muscle fiber development**		
myog	Myog-F	GGACCGAAGCTCCATGGTGGGT
	Myog-R	ACAGTGCTGCTGCTCCTGGTGA
myod	Myod-F	GAGGGCGCCCAGCGGGCATCAT
	Myod-R	ATGATGCCCGCTGGGCGCCCTC
mrf4	Mrf4-F	TGCCTGGAGTGTCCCCACTCTAT
	Mrf4-R	TGCCCATCGCAGTGAGGCTGAAG
mrf5	Mrf5-F	GGACCGCCGGAGAGCTGCCACT
	Mrf5-R	GGATGGCGTTCCTCAGGATCTC
mstna	Mstna-F	GCTGATGAGACTGCATGCCATC
	Mstna-R	GAACGTCGTACTGATCCAGAAG
mstnb	Mstnb-F	CGCAGGAGTGACGTCTTGGCAG
	Mstnb-R	AGTCCATCCTCTCCAGGCTCAG
**FoxO signaling pathways**		
pi3k	Pi3k-F	GTGCTGCTCAAGCTCCTTGAGGT
	Pi3k-R	CCTCAGACTGTCACACAACATCTG
pdk	Pdk-F	GCATGGTGAAGCACTCCATCGGT
	Pdk-R	GACGCAGACTTCCTGGACTCCT
sgk	Sgk-F	GTGGAACTCCTGAGTACTTGGCT
	Sgk-R	TGCGACTGTAGAATGGAGGCAGT
raf	Raf-F	GAATACTGCCCTTCGATATTGAT
	Raf-R	ACCATGTGGACGTTGGGCGTTG
mek	Mek-F	GCCAAGCGCACATACAGAGAACT
	Mek-R	CAGACCTCAACAATATCGTCAAGT
erk	Erk-F	CCCACCTCATGGGAGCAGACCT
	Erk-R	TGAGTGAATATACTTCAGTCCTC
foxo1	Foxo1-F	CATGGTCTACCCAGGCCCTGGGT
	Foxo1-R	CCTGACCCAGGCCCTCCTGTGGA
**Internal contorl**		
18s rRNA	18s-F	TGTCCGAGCGAGCCGTGGAGCT
	18s-R	ATCCAGCATGGCTGTGCTGATG

myog: myogenin, *myod:* myogenic differentiation antigen, *mrf4:* myogenic regulatory factor 4, *mrf5:* myogenic regulatory factor 5, *mstna:* myostatin, *mstnb*: myostatin, *pi3k*: phosphatidylinositol 3-kinase, *pdk*: pyruvate dehydrogenase kinase, *sgk*: serum/glucocorticoid regulated kinase, *raf*: rapidly accelerated fibrosarcoma, *mek:* *m*itogen-activated protein kinase kinase, *erk*: extracellular signal-regulated kinase, *foxo1*: forkhead box O 1.

Table S2 Enriched gene ontology terms associated with muscle development.

GO id	Description	Ratio in study		
		D1 VS D2	D1 VS D3	D2 VS D3
GO:0030239	Myofibril assembly	2/1257	2/620	1/182
GO:0035914	Skeletal muscle cell differentiation	5/1257	6/620	/
GO:0045661	Regulation of myoblast differentiation	/	1/620	/
GO:0045663	Positive regulation of myoblast differentiation	/	1/620	/
GO:0048634	Regulation of muscle organ development	3/1257	2/620	/
GO:0048641	Regulation of skeletal muscle tissue development	3/1257	2/620	/
GO:0048741	Skeletal muscle fiber development	2/1257	/	/
GO:0048747	Muscle fiber development	4/1257	/	2/182
GO:0055001	Muscle cell development	5/1257		2/182
GO:0051147	Regulation of muscle cell differentiation	6/1257	4/620	/
GO:0060537	Muscle tissue development	5/1257	4/620	/
GO:0061061	Muscle structure development	6/1257	5/620	2/182
GO:0007520	Myoblast fusion	2/1257	2/620	/
GO:0090257	Regulation of muscle system process	4/1257	4/620	2/182
GO:1902766	Skeletal muscle satellite cell migration	2/1257	3/620	/
GO:2001014	Regulation of skeletal muscle cell differentiation	2/1257	2/620	/

Table S3 Gene names associated with the Gene id in figure 2.

Gene id	Gene name
gene2014	Titin-like
gene3744	Sarcoplasmic/endoplasmic reticulum calcium ATPase 1
gene4816	Protein arginine methyltransferase 5
gene4867	Rho-related GTP-binding protein RhoA-B
gene7621	Troponin I type
gene7626	Myoblast determination protein 1 homolog
gene8192	Growth/differentiation factor 8
gene8660	Rho-related GTP-binding protein RhoA-D
gene11728	Mechanistic target of rapamycin
gene11867	Rho-related GTP-binding protein RhoA-D
gene12693	Nuclear receptor subfamily 1 group D
gene13405	Dystroglycan-like
gene14387	Paired box protein Pax-7-like
gene14580	Mitogen-activated protein kinase 14B
gene15776	Early growth response 2
gene16433	Eukaryotic translation initiation factor 4E-binding protein 2
gene17651	Homeobox protein SIX4-like
gene19335	Calmodulin
gene20415	RNA binding protein fox-1 homolog 1-like
gene22650	Early growth response protein 2b
gene23300	Ribosomal protein S6 kinase alpha-1
gene23294	Ribosomal protein S6 kinase 2 alpha-like
gene24156	Ryanodine receptor 1-like
gene24359	Transforming growth factor-beta-induced protein ig-h3-like
gene24624	Ephrin type-B receptor 1-like
gene25475	Multiple epidermal growth factor
gene27041	Myosin light chain kinase 2
gene29498	Rho-related GTP-binding protein RhoA
gene30101	Tropomyosin alpha-1 chain
gene33109	Acetylcholine receptor subunit delta
gene35402	Serine/threonine-protein kinase
gene39763	Myogenic factor 5
gene39764	Myogenic factor 4
gene41403	Insulin-like growth factor 1 receptor
gene41941	Calmodulin
gene42287	Troponin T type
gene42462	Protein atonal homolog 8
gene42771	Myogenin-like
gene42925	Alpha-actinin-3-like
gene42929	Alpha-actinin-2-like
gene44152	Myosin-10-like
gene48237	Mitogen-activated protein kinase
gene50754	Kelch-like protein 41a
gene50828	Rho-related GTP-binding protein RhoA-D
gene51570	Junctional adhesion molecule C
gene51604	Insulin-like growth factor II
gene53754	Myristoylated alanine-rich C-kinase
gene54636	Acetylcholine receptor subunit alpha-like
gene60083	Cyclin-dependent kinase inhibitor 1
gene61393	RNA-binding protein 24
gene62133	Rho-related GTP-binding protein RhoA-B
gene62185	Fragile X mental retardation syndrome-related protein 1
gene63842	Histone-arginine methyltransferase
gene64150	Sarcoplasmic/endoplasmic reticulum calcium ATPase 1
gene65575	Dystrophin-like

Table S4 Gene names are associated with the Gene id in the figure 3.

Gene id	Gene name
gene3749	Mitogen-activated protein kinase 1-like
gene4186	Sphingosine 1-phosphate receptor 1-like
gene4706	Serine/threonine-protein kinase B-raf-like
gene5547	G1/S-specific cyclin-D2-like
gene5960	Mitogen-activated protein kinase 1
gene6045	Tumor necrosis factor ligand superfamily member 10-like
gene6320	Insulin-like
gene6658	Phosphatidylinositol 3-kinase regulatory subunit alpha-like
gene7462	Homer protein homolog 1-like
gene7465	Phosphatidylinositol 3-kinase regulatory subunit alpha-like
gene8781	Serine/threonine-protein kinase A-Raf-like
gene10100	NAD-dependent protein deacetylase sirtuin-1-like
gene10643	Mitogen-activated protein kinase 8B-like
gene15683	RAC-gamma serine/threonine-protein kinase-like
gene17813	Forkhead box protein O4-like
gene18589	Transforming growth factor beta-1-like
gene19446	Forkhead box protein O1
gene12648	5'-AMP-activated protein kinase subunit gamma-1-like
gene13838	G2/mitotic-specific cyclin-B1-like
gene20173	F-box protein 32
gene21780	Transforming growth factor beta receptor 2
gene26024	TGF-beta receptor type-2-like
gene27930	Mothers against decapentaplegic homolog 4
gene30678	Krueppel-like factor 2
gene32488	TGF-beta receptor type-1
gene34048	Recombination activating 1
gene38016	Serine/threonine-protein kinase Sgk1-like
gene41403	Insulin-like growth factor 1 receptor
gene41690	Phosphatidylinositol bisphosphate 3-kinase
gene43067	Serine/threonine-protein kinase PLK2-like
gene43117	Insulin receptor-like
gene44712	CREB-binding protein-like
gene46914	F-box only protein 32-like
gene48237	Mitogen-activated protein kinase 14B
gene52353	Forkhead box protein O3-like
gene55385	Serine/threonine-protein kinase NLK-like
gene58506	Mitogen-activated protein kinase 9-like
gene60083	Cyclin-dependent kinase inhibitor 1-like
gene60477	Signal transducer and activator of transcription 3-like
gene61202	Signal transducer and activator of transcription 1-alpha/beta-like
gene62883	Gamma-aminobutyric acid receptor-associated protein-like 1
gene64275	GTPase NRas-like

gene65510	Serine/threonine-protein kinase PLK3-like

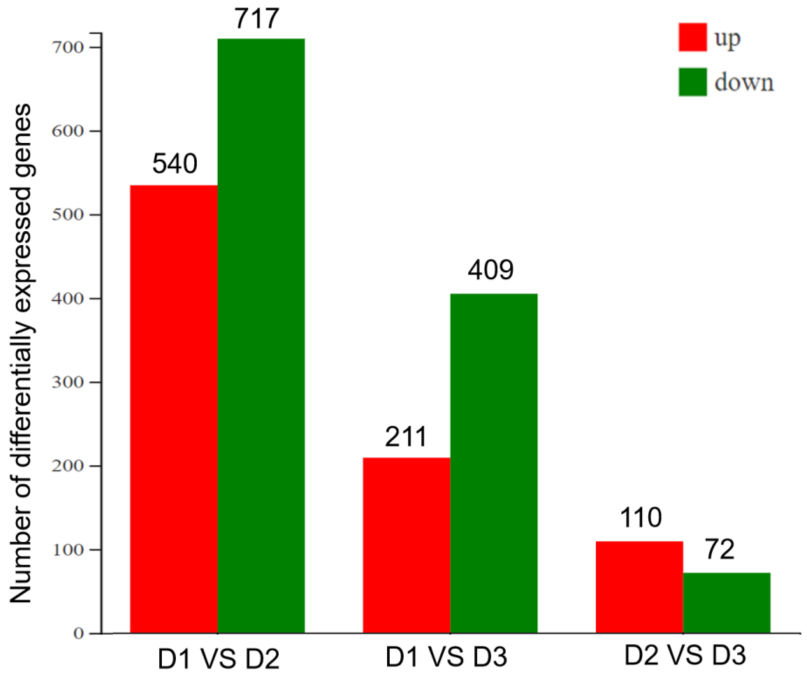

Figure S1 Number of differentially expressed genes showing upregulated or downregulated expression among D1, D2, and D3 groups.

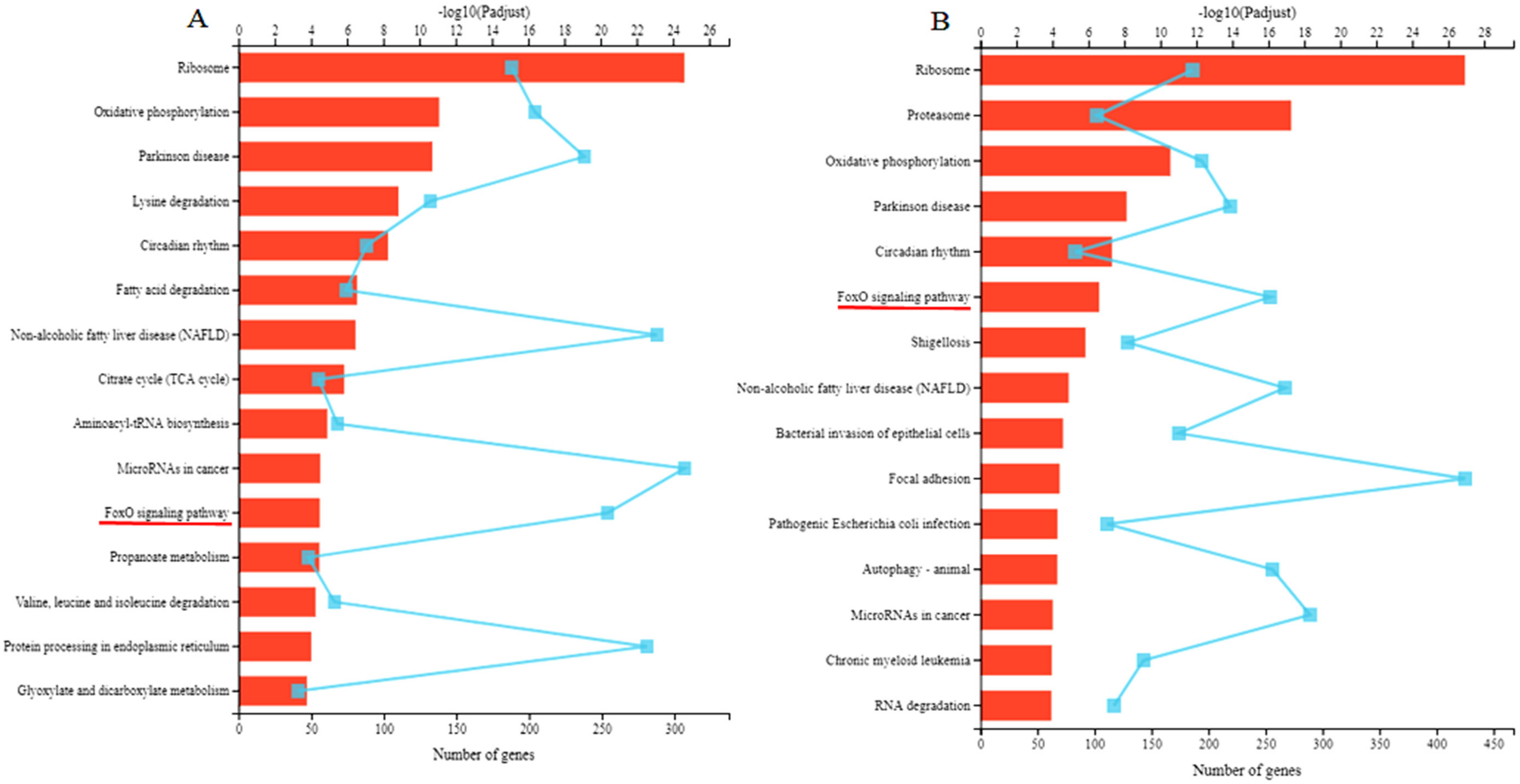

Figure S2 The top 20 GO categories of the differential genes among D1 versus D2(A), and D1 versus D3(B)

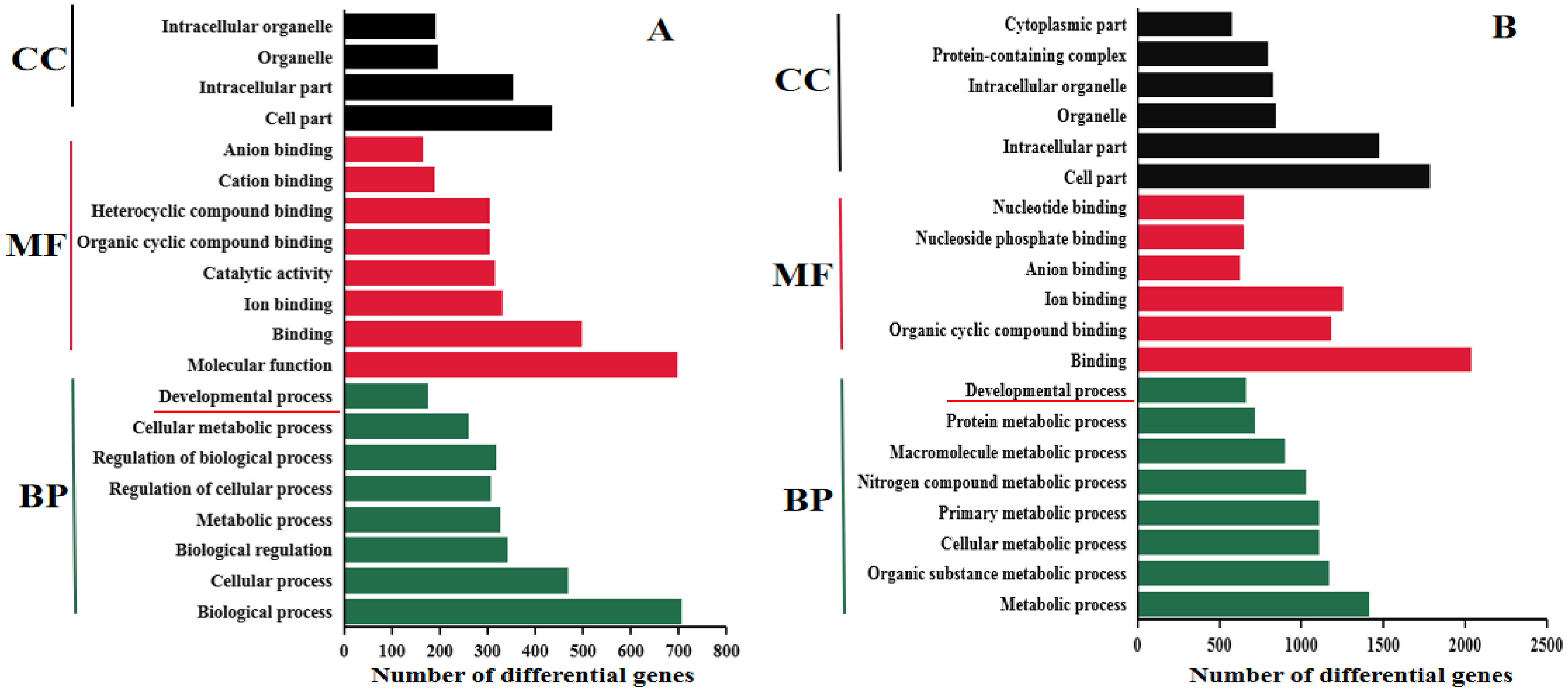

Figure S3 The top 15 significantly enriched the KEGG pathway of the differential genes between D1 versus D2 (A), and D1 versus D3 (B).

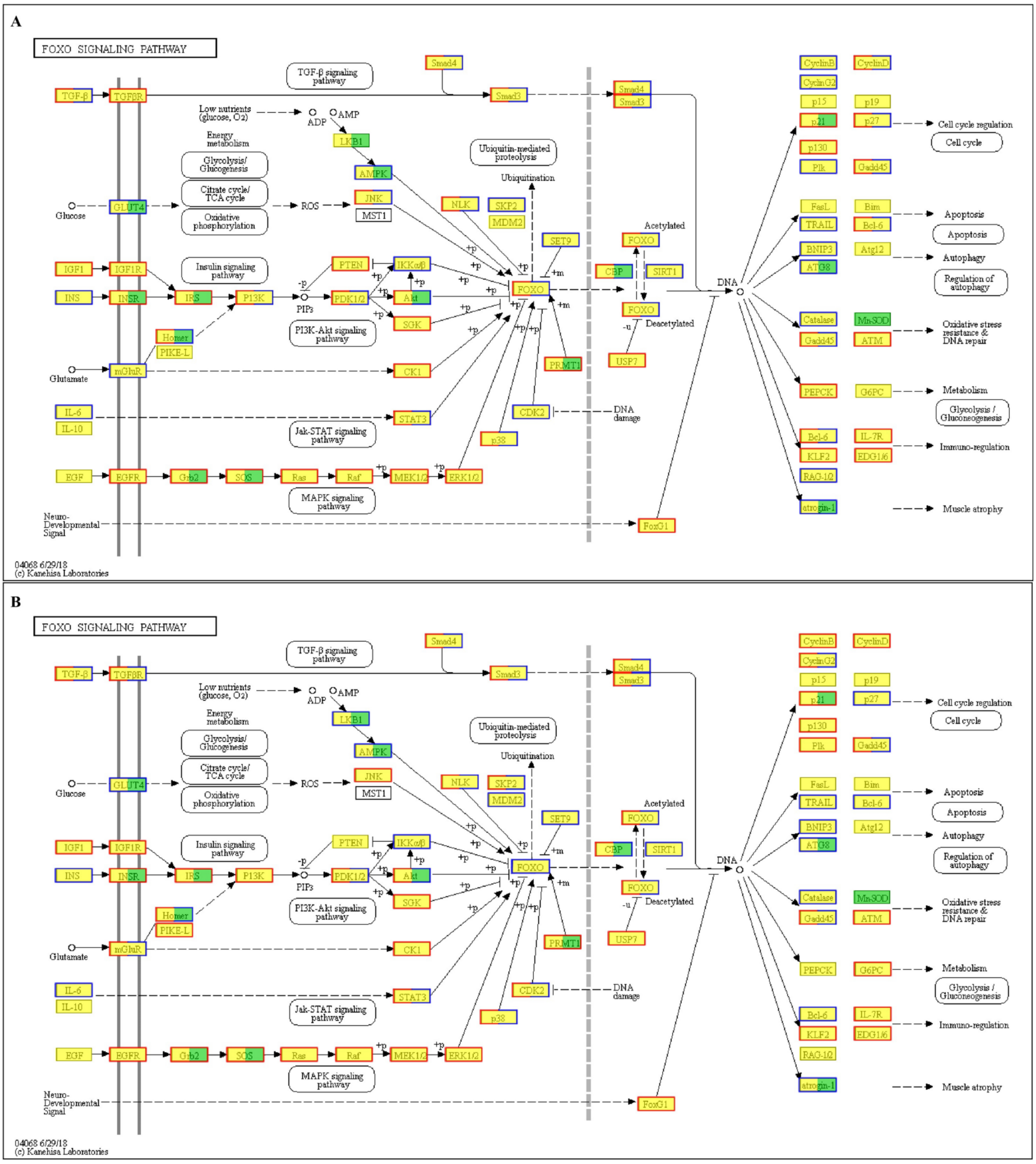

Figure S4 FoxO signaling pathways. (A) Differentially expressed transcripts between D1 and D2. (B) Differentially expressed transcripts between D1 and D3. The red color indicates up-regulation, the blue color indicates down-regulation, and half red and half blue indicate up+ down regulation.
